# A highly quality genome sequence of *Penicillium oxalicum* species isolated from the root of *Ixora chinensis* in Vietnam

**DOI:** 10.1093/g3journal/jkac300

**Published:** 2022-12-01

**Authors:** Huong Mai Pham, Dung Thi Le, Lam Tung Le, Phuong Thi Minh Chu, Linh Huyen Tran, Tung Thanh Pham, Hung Mau Nguyen, Tien Thuy Luu, Ha Hoang, Hoang Ha Chu

**Affiliations:** Institute of Biotechnology (IBT), Vietnam Academy of Science and Technology (VAST), Hanoi 10072, Vietnam; Institute of Biotechnology (IBT), Vietnam Academy of Science and Technology (VAST), Hanoi 10072, Vietnam; Institute of Biotechnology (IBT), Vietnam Academy of Science and Technology (VAST), Hanoi 10072, Vietnam; Institute of Biotechnology (IBT), Vietnam Academy of Science and Technology (VAST), Hanoi 10072, Vietnam; Institute of Biotechnology (IBT), Vietnam Academy of Science and Technology (VAST), Hanoi 10072, Vietnam; Institute of Biotechnology (IBT), Vietnam Academy of Science and Technology (VAST), Hanoi 10072, Vietnam; Institute of Biotechnology (IBT), Vietnam Academy of Science and Technology (VAST), Hanoi 10072, Vietnam; Institute of Biotechnology (IBT), Vietnam Academy of Science and Technology (VAST), Hanoi 10072, Vietnam; Institute of Biotechnology (IBT), Vietnam Academy of Science and Technology (VAST), Hanoi 10072, Vietnam; Institute of Biotechnology (IBT), Vietnam Academy of Science and Technology (VAST), Hanoi 10072, Vietnam; Graduate University of Science and Technology, Vietnam Academy of Science and Technology (VAST), Hanoi 10072, Vietnam

**Keywords:** *Penicillium*, fungal genome, chromosome-level assembly, long-read sequencing, reference genome

## Abstract

*Penicillium oxalicum* has been reported as a multienzyme-producing fungus and is widely used in industry due to great potential for cellulase release. Until now, there are only 10 available genome assemblies of *P. oxalicum* species deposited in the GenBank database. In this study, the genome of the I1R1 strain isolated from the root of *Ixora chinensis* was completely sequenced by Pacbio Sequel sequencing technology, assembled into 8 chromosomes with the genome size of 30.8 Mb, as well as a mitogenome of 26 kb. The structural and functional analyses of the I1R1 genome revealed gene model annotations encoding an enzyme set involved in significant metabolic processes, along with cytochrome P450s and secondary metabolite biosynthesis. The comparative analysis of the *P. oxalicum* species based on orthology and gene family duplications indicated their large and closed pan-genome of 9,500 orthologous groups. This is valuable data for future phylogenetic and population genomics studies.

## Introduction

*Penicillium*, a genus of ascomycetous fungi, first described by Link (1809), contributes significant parts in both the natural environment and human research and production activities. One of the most well-known compounds produced by some members of this genus is penicillin, discovered by Alexander Fleming (1929). Among hundreds of species of the *Penicillium* genus, many are involved in the decomposition saprophytes of organic materials. Several reports show that some *Penicillium* spp. can display negative impacts to the food crop as pathogens (Frisvad and Samson 2004; Pitt and Hocking 2009; Samson *et al*. 2010) while some can enhance plant growth (Khan *et al*. 2008; Byrappa *et al*. 2011). Application of *Penicillium* spp. can also be found in the food industry, such as cheese (Charles 1906; Nelson 1970; Karahadian *et al*. 1985; Giraud *et al*. 2010) and fermented sausages production (López-Díaz *et al*. 2001; Ludemann *et al*. 2010). To date, 30 *Penicillium* genomes among 18 species/483 accepted species were reported. The most complete genome is from Penicilina (Yin *et al*. 2021).

*Penicillium oxalicum* (previously called *P. decumbens*) is an anamorphic species of the genus *Penicillium*, which was named based on the feature of oxalic acid production (Currie 1915). This ubiquitous fungus can be found in various environments including soil, air, food, and tropical communities (Currie 1915; Pitt and Hocking 2009; Gusareva *et al*. 2019; Gupta *et al*. 2020). Apart from the oxalic acid production, there are evidences that some strains of *P. oxalicum* species are capable of releasing several bioactive compounds related to antifungal (Yang *et al*. 2008), antimicrobial characteristics (Shuo *et al*. 2014), as well as antitumor activities in human cell lines (Shuo *et al*. 2014; Ding *et al*. 2020). It was also reported to be used to remediate toxic metals like chromium and lead (Long *et al*. 2018; Tian *et al*. 2019; Hao *et al*. 2021). *P. oxalicum* is well known as a multienzyme-producing fungus producing cellulase, lignocelluloses, xylanases, and glycoside hydrolases. *P. oxalicum* exhibits a great potential for cellulase release to hydrolyze lignocellulose and has been used in industrial cellulase production (Liu *et al*. 2013; Yao *et al*. 2015; Li *et al*. 2016). *P. oxalicum* also contains some of the unique enzymes for lignocellulase degradation and is more balanced and effective than the enzyme system from *Trichoderma reesei* (Gao *et al*. 2011; Ma *et al*. 2011; Liu *et al*. 2013). However, a comprehensive understanding of the enzyme system in this fungal species is still quite limited due to a lack of genetic information.

In this study, we present the de novo sequencing of the whole genome of *P. oxalicum* I1R1. This strain was isolated from the root of *Ixora chinensis*, whose flower extraction was reported to have ability of cancer inhibition, and deposited in glycerol (50% v/v), at temperature of −80°C, at the National Key Laboratory of Gene Technology (IBT-VAST, Hanoi, Vietnam). The *P. oxalicum* I1R1 assembly was evaluated in comparison with other published fungal genomic sequences belonging to the *P. oxalicum* species. Annotation was also examined to provide valuable information for efforts to determine the potential functions of *P. oxalicum*.

## Materials and methods

### Genomic DNA preparation

The *Penicillium* sp. I1R1 strain isolated from the root of *I. chinensis* was cultured on Potato dextrose agar (PDA) media at 28°C ± 2 from glycerol stock and used for DNA extraction after 14 days of cultivation. I1R1 high-molecular-weight genomic DNA was collected by using Zymo Research Quick-DNA Fungal/Bacterial Miniprep Kit according to the manufacturer's instructions. A total 100 mg sample was used for each extraction. Before being used as a template for DNA library preparation, DNA product was quantified via 0.8% agarose gel electrophoresis using Pippin pulse—SAGE, Qubit dsDNA HS Assay Kits by QUBIT 3.0, and taxonomical reconfirmed through amplification of ITS1-5.8S-2 region and Sanger sequencing.

### Library preparation and genomic sequencing

A total of 500 ng of I1R1 genomic DNA diluted in 150 µl Elution Buffer buffer was sheared by the g-TUBE (Covaris) into small fragments of 10 kb, and checked via Bioanalyzer Agilent 2100 with Agilent DNA 12000 Kit (5067-1508 Agilent). The sequencing library was prepared by SMRTbell Express Template Prep Kit 2.0 (100-938-900; PacBio), followed by polymerase attached and purification by the Sequel Binding and Internal Ctrl Kit 3.0 (101-626-600; PacBio). Calculation and setting up for loading on Sample Plate (000-448-888; PacBio) with genomic DNA (concentration of 9 pM) was performed on Sample Setup software in SMRTLink portal version 9.0. The fungal genome was sequenced by PacBio SEQUEL with the Sequel SMRT Cell 1 M v3 Tray chip (101-531-001; PacBio) and Sequel Sequencing Kit 3.0 (101-597-900; PacBio) at the Institution of Biotechnology, Vietnam Academy of Science and Technology (Hanoi, Vietnam).

### Genome assembly, prediction, and annotation

The PacBio long reads from Single Molecule Real-Time (SMRT) DNA sequencing were assembled using the hierarchical genome-assembly process version 4 for high-quality de novo fungal genome assembly (Chin *et al*. 2013). Telomeric repeats were identified by the FindTelomeres python script (https://github.com/JanaSperschneider/FindTelomeres). Graphic representation of the I1R1 assembled chromosomes was visualized via the software Geneious Prime version 2021.2.2 (https://www.geneious.com/). Whole-genome alignment between *P. oxalicum* I1R1 and the reference strain CCTCC M 20211203 (GenBank assembly accession: GCA_021133555.1) was conducted and visualized by MAUVE (Darling *et al*. 2010). Analysis of synteny between I1R1 and CCTCC M 20211203 was carried out using Sibelia version 3.0.7 (Minkin *et al*. 2013) and identified syntenic regions were visualized via Circos software (Krzywinski *et al*. 2009). Regarding structural annotation, repeat sequences were first masked from the *P. oxalicum* I1R1 genome using the RepeatModeler version 2.0.2 (Flynn *et al*. 2020) and RepeatMasker version 4.1.2 programs (http://www.repeatmasker.org). Long read mapping to the reference *Penicillium citrinum* mitochondria (NCBI Reference Sequence: NC_047444.1) was accomplished using pbmm2 (PacificBiosciences 2021) and the I1R1 mitochondrial genome was then assembly by Unicycler (Wick *et al*. 2017). Protein-coding sequences were predicted using the automated pipeline BRAKER2 (Brůna *et al*. 2021), a combination of GeneMark-ET (Brůna *et al*. 2020) and AUGUSTUS (Stanke *et al*. 2006), in which we used I1R1 genome file and file with proteins of short evolutionary distance genomic from *Penicillium chrysogenum* reference genome ASM71027v1 (GenBank: GCA_000710275.1) to automatically generate full gene structure annotations in novel genomes. The sequences of ribosomal RNAs (rRNAs) were identified by barrnap prediction software version 0.9 through the whole-genome sequence (Seemann 2018). The genes encoding transfer RNAs (tRNAs) were predicted by tRNAscan-SE software version 2.0 (Chan *et al*. 2021). A final analysis was then generated using BUSCO software version 5.2.2 using the eurotiales_odb10 gene set to verify the completeness of the genome assembly and protein annotation (Simão *et al*. 2015).

The functional annotations of predicted protein-coding sequences were performed through orthology assignment by eggNOG-Mapper version 2 (Cantalapiedra *et al*. 2021), in which, protein models were classified according to Gene Ontology (GO), Eukaryotic Orthologous Groups (KOG), Kyoto Encyclopedia of Genes and Genomes (KEGG), and Pfam data. The GO terms were then analyzed for tree hierarchical classification using the WEGO web tool (https://wego.genomics.cn/; Ye *et al*. 2018). The GO annotations include 2 layers describing the role of the protein in biological functions. The KOG annotations were classified into functional groups based on the Cluster of Orthologous Groups (COG) database. KEGG terms are divided into 4 levels with the third and fourth levels being the specific biosynthetic pathways and genes regulated in each pathway, respectively. Annotations of carbohydrate-active enzymes (CAZymes) for the *P. oxalicum* I1R1 genome were performed using the CAZy database by dbcan2 meta server, with diamond and hmmscan at https://bcb.unl.edu/dbCAN2/ (Zhang *et al*. 2018). For comparative genomic analysis, the predicted proteomes of the I1R1 strain with other *Penicillium* species were used for pangenomic analysis through predicting orthologous genes and gene family duplications using the orthoMCL tool (Li *et al*. 2003). The groups with at least one copy in each genome were considered core orthologous groups. Secondary metabolite biosynthetic gene clusters (smBGCs) in the *P. oxalicum* I1R1 genome were annotated by the antiSMASH version 6.1.1 (Blin *et al*. 2021). The NRPS/PKS domains of the core biosynthetic genes with functional motifs/sites were visualized in DOG (Domain Graph) software version 2.0 (https://dog.biocuckoo.org/). The mitochondrial annotation was executed via Mitos webtool version 2 (Donath *et al*. 2019), and the MFannot and RNAweasel pipelines available at https://megasun.bch.umontreal.ca/. The circular mitochondrial genome map was then visualized through the webtool OrganellarGenomeDRAW (OGDRAW; Lohse *et al*. 2007). The multiple mitogenome alignment was executed using LAGAN mode in mVISTA web-based tool (Frazer *et al*. 2004).

### Phylogenetic analysis

We sequenced the ITS1-5.8S-ITS2 region of the I1R1 strain by the primer ITS4/ITS5 for standard fungal taxonomy. We extracted ITS sequences by ITSx (Bengtsson-Palme *et al*. 2013) with default parameters and plus “–save-regions all” to get SSU, 5.8S, LSU sequences. To affirm the taxonomic position of the new strain, we construct phylogenetic analysis based on both complete mitochondrial genome sequences and ITS sequences. Thirteen mitochondrial complete sequences were used to build the maximum-likelihood phylogenetic tree. The following mtDNAs were selected to construct the tree: *Aspergillus parasiticus* (NC_041445.1), *Aspergillus oryzae* (NC_008282.1), *Aspergillus fumigatus* (NC_017016.1), *Aspergillus niger* (NC_007445.1), *Aspergillus tubingensi* (NC_007597.1), *Aspergillus kawachii* IFO 4308 (AP012272.1), *Aspergillus nidulans* FGSC A4 (JQ435097.1), *Penicillium* sp. ShG4C (KX931017.1), *P. citrinum* (MK919205.1), *Penicillium polonicum* (KU530219.1), *Penicillium digitatum* strain pd01 (HQ622809.1), and *Cladosporium sphaerospermum* (NC_050879.1). Multiple alignment of those sequences was performed using the MAFFT online tool (https://mafft.cbrc.jp/alignment/server/; Katoh *et al*. 2002) with default parameters. Approximately, maximum-likelihood phylogenetic trees were then constructed based on generalized time-reversible models of nucleotide evolution by FastTree version 2.1 (Price *et al*. 2010), and visualized by FigTree version 1.4.4 (http://tree.bio.ed.ac.uk/software/figtree).

## Results and discussion

### Sequencing and prediction data

**Fig. 1.**
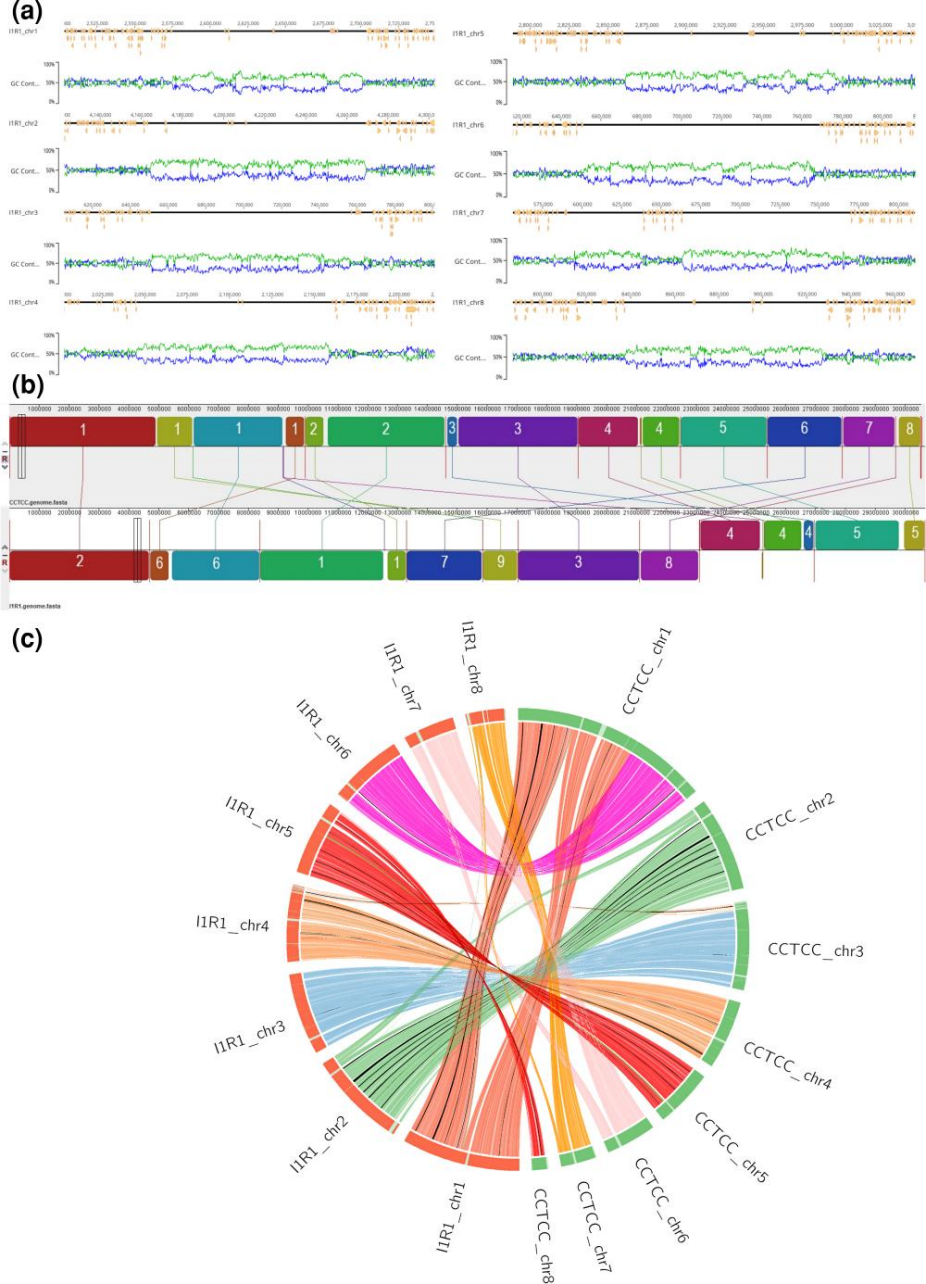
Genome features of the chromosome-scale assembly the *P. oxalicum* I1R1 genome. a) Graphic representation of the 8 *P. oxalicum* I1R1–assembled chromosomes containing centromeric regions. b) Whole-genome alignment between the I1R1 strain and the reference CCTCC M 20211203 strain. The blocks represent the LCBs of the 2 genomes and the numbers within blocks are the chromosomes which these LCBs belong to. c) Syntenic regions between the I1R1 and CCTCC M 20211203 scaffolds.

The details of the structural annotation summary statistics of *P. oxalicum* I1R1 are presented in Table 1. The assembly process initially resulted in 9 contigs whose total consensus genome size of 30.8 Mb. The largest and smallest contig sizes are 4.91 and 1.19 Mb, respectively. Six of 9 contigs are represented by complete telomere-to-telomere assemblies at chromosome-level (Supplementary Table 1). We identified 15 out of 18 telomeres and it was not possible to complete the assembly of the 3 remaining contigs due to the de novo approaching limitations. Each chromosome was flanked by 16–18 telomeric repeats “TTAGGG/CCCTAA” that were similar to those identified in other *Penicillium* species (Cardoso *et al*. 2007; Hao *et al*. 2021). The centromeric regions within filamentous fungal genomes commonly are ∼100 kb of length, have high AT content, and typically lack coding regions (Smith *et al*. 2012). These criteria were used to identify putative centromeric regions in the assembled *P. oxalicum* I1R1 genome and predicted regions were over 100 kb. In addition, several contigs possessed additional AT-rich regions surrounding the predicted centromeric regions (Fig. 1a). However, there was no region satisfying the above criteria in contig 9 (the shortest contig of 1.91 Mb); thus, the centromere was not identified in this contig. These findings of functional centromeric regions of the I1R1 genome were based on the prediction of bioinformatic approach; thus, the verification would be required by executed further experimentation, for instance, the identification of a centromere identifier—the specialized histone H3 variant (Smith *et al*. 2011; Yadav *et al*. 2018). The whole-genome alignment of I1R1 and the reference strain CCTCC M 20211203 (GenBank assembly accession: GCA_021133555.1) by progressiveMAUVE algorithms revealed the locally collinear blocks (LCBs) between the two genomes (Fig. 1b). Interestingly, the first chromosome of the reference genome showed several LCBs with three contigs of the I1R1 genome (contigs 2, 9, and 6; in that order across the sequence). Along with the results from the telomeric and centromeric identifications of the second and ninth I1R1 contigs, we combined these two contigs into a chromosome. However, there was a gap of “NNN” between the two partial contigs present in this chromosome, corresponding to the region from 4,924,407 to 4,927,159 bp in the reference's first chromosome, that cannot be assembled in the present study due to the lack of mapping reads. This suggested that the genome size of this fungal strain could be >30.8 Mb. Regarding the sixth I1R1 contig, it was a completely assembled chromosome with predicted telomeres at each end as well as a putative centromere (Supplementary Table 1). Applying a similar approach, we verified the 6 remaining I1R1 contigs as 6 chromosomes. The finished assembly of the I1R1 genome consisted of 8 chromosomes with 8 centromeres and 15 out of 16 telomeres identified (see detail in Supplementary Table 1).

**Table 1.** The characteristics of the assembly genome of *P. oxalicum* strain I1R1.

Parameters	Values
Size (∼Mb)	30.8
No. of chromosomes	8
Contig N50	3,834,910
Contig L50	4
Coverage	400
Completeness (%)	95
GC content (%)	50.6
tRNA genes	421
rRNA genes	55
No. of protein-coding genes	8,317
Average gene length (bp)	1,731.54
Average exon length (bp)	543.4494494
Average no. of exons per gene	3
Total repeat length (bp)	1,502,712 (4.88%)

*Penicillium oxalicum* I1R1 was analyzed for synteny with *P. oxalicum* CCTCC M 20211203 based on their respectively assembled scaffolds. The two genomes comprised different numbers of chromosomes but this analysis compared the length of unambiguously assembled scaffolds for homologous scaffolds. Syntenic regions between I1R1 and CCTCC M 20211203 were identified after aligning 30.8 Mb of assembled sequences from I1R1 with CCTCC M 20211203 scaffolds (Fig. 1c). A total of 1457 one-to-one matching scaffold block pairs ranging from 499 bp to 193.6 kb were identified between the two genomes. There are 1,418 blocks with 2 instances, and they cover 94.51% of all 17 chromosomes. All the blocks cover 95.11% of all the input sequences. The CCTCC M 20211203s ninth chromosome showed no syntenic region compared with the I1R1 genome due to the absence of homologous scaffolds.

The assembly of *P. oxalicum* strains I1R1 has the Contig N50 value of 3.83 Mb and the Contig L50 value of four. The prediction of ab initio genes using the automated pipeline BRAKER2 shows that the *P. oxalicum* I1R1 genome includes 8,317 protein-coding genes. One hundred and fifty-three genes were predicted to encode hypothetical proteins. The average size of protein-coding genes is 1,731 bp with averages of 3 exons per gene. For RNA, 421 tRNAs and 55 rRNAs were predicted. According to the available genome assemblies of different *P. oxalicum* strains in DDBJ/ENA/GenBank (Supplementary Table 2), the quality of our set of whole-genome de novo sequencing, assembly, and annotation data is currently the most complete genome-scale and high-quality assembly. To our knowledge, there are only 8 available genome assemblies of *P. oxalicum* species from ten strains (namely I1R1, CCTCC M 20211309, SGAir0226, HP7-1, SYJ-1, 114-2, JU-A10-T, YT02, PM4501B, and M7025A) deposited to the database. The genome sizes vary from 29.83 to 31.25 Mb. Different sequencing technologies greatly affect the number of generated contigs after assembly (Miyamoto *et al*. 2014). Assemblies from short-reads sequencing platforms such as Illumina HiSeq, Illumina GAIIx, ABI SOLiD, and 454 GS-FLX Titanium contain more contigs (from 176 contigs to 674 contigs) while assemblies from long-read sequencing platforms such as PacBio and Oxford Nanopore PromethION results in fewer contigs (8 contigs in the assembly of strain I1R1 and HP7-1, 9 contigs in CCTCC M 20211309, and 20 contigs in SGAir0226). Most of the available genomes have a GC content of 50.6%, including *P. oxalicum* I1R1. BUSCO analysis (Supplementary Table 2) using the eurotiales_odb10 gene set (*n* = 4191) shows that the genome assembly of I1R1 has the gene space completeness of 95% which represents high-quality assembly, similar to the data of 94.9% of the representative species *P. oxalicum* HP7-1. The new I1R1 assembly was equal in size to the HP7-1 assembly with 4.88% of the total-genome sequence being the repetitive regions (Table 1).

### Mitochondrial genome and phylogenetic analysis

**Fig. 2.**
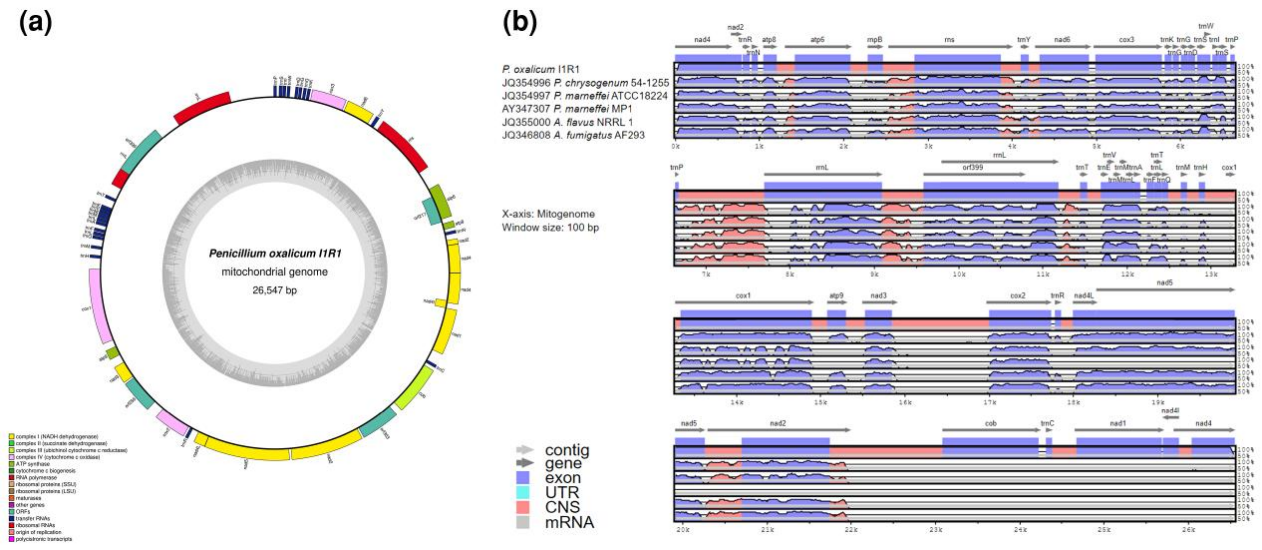
The circular mitochondrial genome and comparison visualization of the *P. oxalicum* I1R1. a)The circular map shows the core genes as part of the NADH dehydrogenase, ubiquinol cytochrome c reductase, cytochrome c oxidase, and ATP synthase protein complexes. The RNAseP subunit (rnpB), small and large rRNAs (rns and rnl), as well as tRNAs are also displayed. Additional ORFs are shown along the circular map. b) The identity plot comparing the mitogenomes of I1R1 with 3 other *Penicillium* species and 2 *Aspergillus* species.

According to the GenBank database, the previously published *Penicillium* mitochondrial assemblies commonly suggested 26–28 kb circular mapping genomes. Regarding I1R1 strain, the Unicycler assembly of reads only aligning to the reference mitochondrial sequence showed that the mitogenome is 26,547 bp and confirmed that it is circular (Fig. 2a). The annotation revealed a total of 17 protein-coding mitochondrial genes as part of the NADH dehydrogenase, ubiquinol cytochrome c reductase, cytochrome c oxidase, and ATP synthase protein complexes, along with 4 additional ORFs. One large and one small rRNAs (rnl and rns), one RNAseP subunit (rnpB), as well as 27 tRNAs were also annotated. The previous study on the core genome analysis of 15 *Aspergillus* and *Penicillium* mitochondrial genomes (Joardar *et al*. 2012) resulted in 14 core protein-coding genes, all present on the forward strand. We executed the multiple alignment of the I1R1 mitogenome with 5 other mitochondrial genomes. These above core genes were also predicted in the I1R1 mitogenome with conservative gene order (Fig. 2b).

**Fig. 3.**
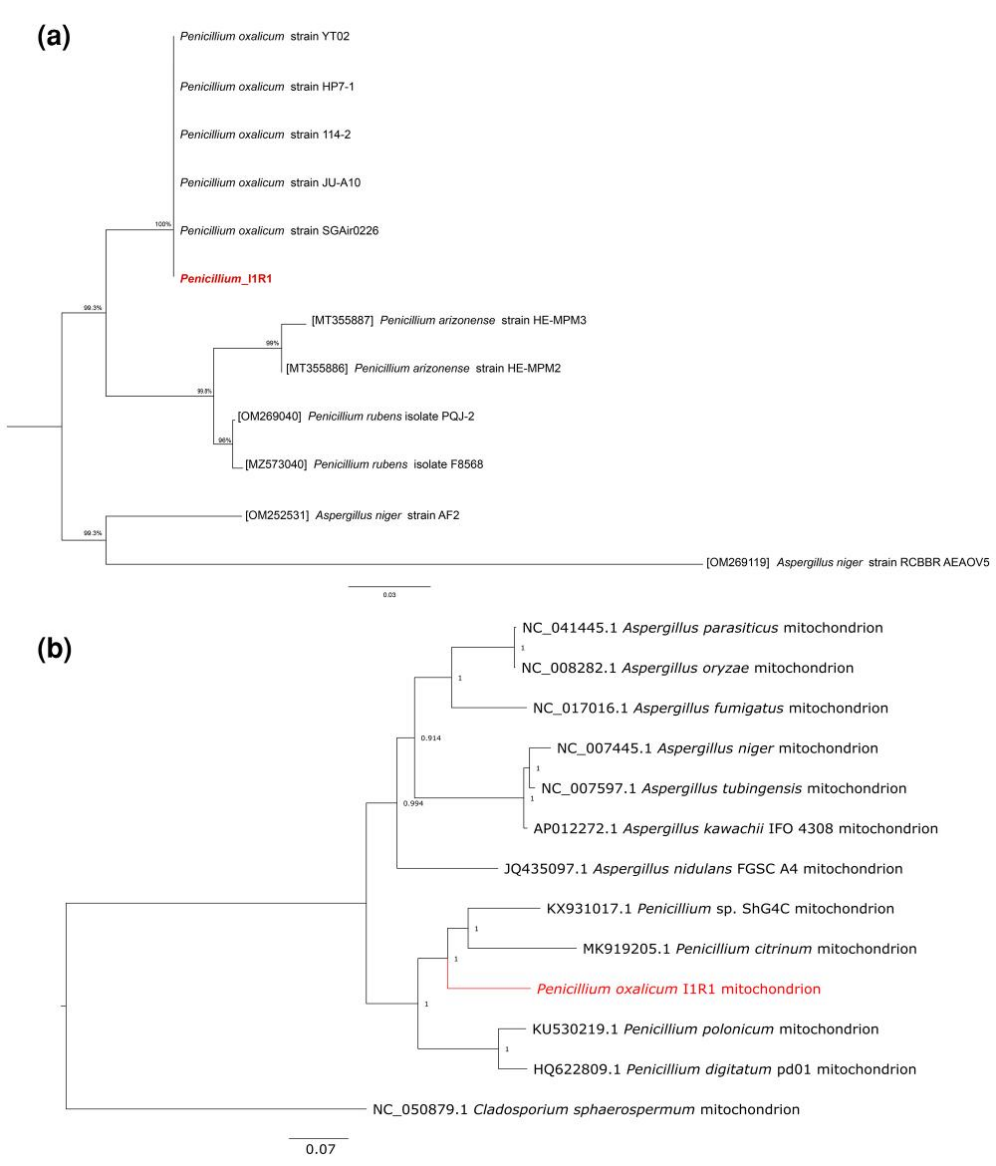
Bootstrap maximum-likelihood phylogenetic trees of *Penicillium* sp. based on ITS region sequences (a) and complete mitogenome (b). The graph shows clustering I1R1 with *P. oxalicum*.

A phylogenetic tree based on the ITS1-5.8S-ITS2 region sequences with additional reference *Penicillium* species was constructed to investigate the phylogenetic relations of *P. oxalicum* I1R1. The I1R1 belongs to the *P. oxalicum* species with a bootstrap value of 100% for support (Fig. 3a). Besides, the phylogenetic tree based on the mtDNAs verified affiliation of the strain I1R1 with the genus *Penicillium*, where it formed a separated species-level clade with other strains (ShG4C and citrinium). A high bootstrap value of 100 supports all branches of this clade (Fig. 3b). Numbers at nodes indicate bootstrap support values from 1,000 replicates. The results confirmed taxonomical classification and morphological characterization in the *P. oxalicum* I1R1 with colony presence white, shades of pineapple punch, circular form, raised elevation, and entire margin after 7-day growth on PDA medium. Additionally, I1R1 showed features belonging to *Penicillium* species by conidia and branching pattern characters (Supplementary Fig. 1).

### Functional annotation

The number of genes annotated by different public databases KOG, KEGG pathway, and GO are 7,226, 4,465, and 3,840, respectively (Table 2).

**Table 2.** Functional annotation of *P. oxalicum* I1R1 genes from different public databases.

No. of genes annotated by public protein database	No. of genes	Percentage
No. of protein-coding genes	8,317	100
No. of genes annotated by KOG	7,226	86.88
No. of genes annotated by KEGG pathway	3,805	45.7
No. of genes annotated by GO	3,840	46.17

The COG analyses were performed to predict the functional classification of all genes. A total of 7,226 genes of the *Penicillium oxalicum* I1R1 genome were involved in 25 KOG categories (Supplementary Fig. 2a). Among them, the largest group was the “S: Function unknown” (2,027 genes, 28.05%), followed by “G: Carbohydrate transport and metabolism” (506 genes, 7%), O: Post translational modification, protein turnover, chaperones (476 genes, 6.59%), and “E: Amino acid transport and metabolism” (441 genes, 6.1%; Supplementary Fig. 2a). For GO analysis, 3,840 genes, accounting for 46.17% of total annotated genes, were assigned the 3 general GO categories: cellular component, molecular function, and biological process (Supplementary Fig. 2b, Supplementary Table 3). In total, 72 subcategories were subdivided from the GO terms: 16, 23, and 33 subcategories for cellular component, molecular function, and biological process, respectively. Cell/cell part, organelle, and cellular processes were the most abundant subcategories. Functional annotation of the complete genome sequence of *P. oxalicum* I1R1 reveals its broad abilities to grow, transport, metabolize, and respond to different environments, as well as its capabilities of exporting compounds such as enzymes.

In order to understand the biological functions and gene interactions of *P. oxalicum* I1R1, an annotation of the KEGG pathway database was performed and identified 3,805 genes (accounting for 45.7% of total predicted genes) assigned into 6 levels, including 45 KEGG pathways (Supplementary Table 4). Of the four main levels, metabolism such as carbohydrate metabolism was the largest (1,396, 36.69%), followed by genetic information processing such as translation (836, 21.97%), cellular processes such as signal transduction (661, 17.37%), and environmental information processing such as transport and catabolism (436, 11.46%; Supplementary Fig. 3). Thus, the majority of genes in the I1R1 genome correspond to significant metabolic processes.

The cytochrome P450 (CYP) superfamily consists of enzymes involved in a variety of physiological processes, including xenobiotic degradation, detoxification, and biosynthesis of secondary metabolites (Črešnar and Petrič 2011; Yap *et al*. 2014). KEGG analysis revealed that *P. oxalicum* I1R1 has 5 genes classified as “Drug metabolism—cytochrome P450” and 5 genes classified as “Metabolism of xenobiotics by cytochrome P450” (Supplementary Table 5). Compared with the majority of other fungi, *P. oxalicum* I1R1 has few P450 genes involved in KEGG pathways.

The pan-genome analysis resulted in 9,500 groups in *P. oxalicum* genomes, of which 5,203 and 4,297 were core and accessory orthologous groups for the 10 fungal strains tested, respectively. The size of the pan-genome grew steadily with the addition of each further genome showing that *P. oxalicum* possesses a large pan-genome. The number of accessory orthologous groups varied greatly between strains, ranging from 1,827 for *P. oxalicum* SGAir022 to 3901 for *P. oxalicum* HP7 (Table 3). The core orthologous groups account for a major proportion; thus, *P. oxalicum* has a closed pan-genome. Among the 5,203 core orthologous groups clustered, 150 were annotated to pfam domains and 5,053 were single-copy gene orthologs present in all fungi, suggesting these may be strain-essential genes. There were 4,447 singleton genes (strain-specific genes) among the *P. oxalicum* strains, consisting of 106 specific gene models belonging to the *P. oxalicum* I1R1 genome. Orthologous analysis is the most accurate approach to investigate the similarities and differences between model organisms in order to infer the functional genetic information and propose function in novel sequenced genomes (Gabaldón and Koonin 2013).

**Table 3.** Pan-genome statistics of *P. oxalicum* strains.

*P. oxalicum* strains	Input protein sequences	Accessory orthologous groups	Singletons
CCTCC	7184	1981	705
114	7908	2705	1939
SGAir0226	7030	1827	872
SYJ1	7635	2432	255
YT02	7909	2706	18
I1R1	8317	2852	106
PM4501B	7865	2662	14
HP7	9104	3901	478
M7025A	7861	2658	20
JUA10T	8044	2841	20

### The carbohydrate-active enzyme family

**Fig. 4.**
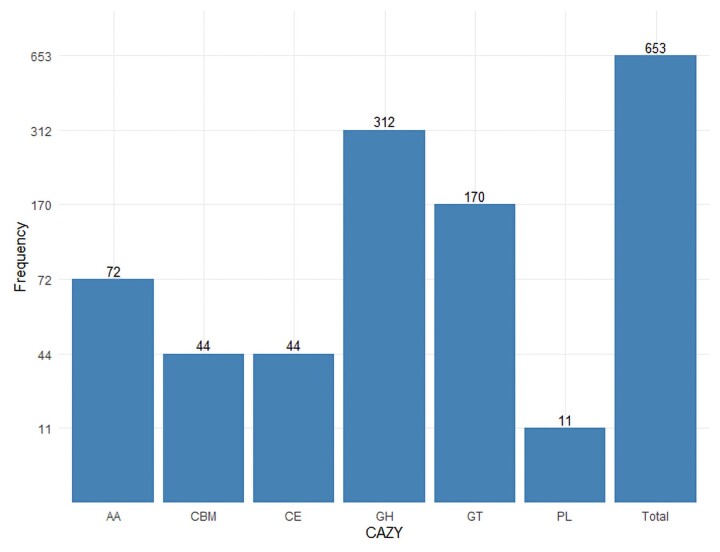
Carbohydrate-active enzyme (CAZyme) analysis results of *P. oxalicum* I1R1 genome. A total of 653 genes encoding CAZymes include glycoside hydrolases (GHs), auxiliary activities (AAs), carbohydrate-binding modules (CBMs), carbohydrate esterases (CEs), glycosyl transferases (GTs), and polysaccharide lyases (PLs). The *y*-axis shows the number of protein-coding genes in each CAZy term.

The *Penicillium oxalicum* I1R1 genome sequence was used to determine the presence of CAZymes. The analysis predicted 653 genes encoding CAZymes includes 312 glycoside hydrolases (GHs), 72 auxiliary activities (AAs), 44 carbohydrate-binding modules (CBMs), 44 carbohydrate esterases (CEs), 170 glycosyl transferases (GTs), and 11 polysaccharide lyases (PLs; Fig. 4). The predictions suggest more GHs than GTs in the *P. oxalicum* I1R1 genome. Analysis of the CAZy database revealed the I1R1 strain encodes a plentiful set of CAZymes in abundance and distribution, similar to those of other *Penicillium* fungi. The I1R1 genome appears to encode a large number of enzymes involved in the degradation of plant cell–wall polysaccharides, such as, hemicellulose (145), cellulose (107), and pectin (100; Supplementary Table 6). These observations, combined with the KEGG pathway results, reconfirm the potential of *P. oxalicum* I1R1 for cellulase degradation. In addition, the I1R1 strain seems capable of degrading non-plant polysaccharides (for instance, bacterial and animal polysaccharides), due to the presence of CAZymes in GH79, GH88 families (Supplementary Table 6). A wide range of CAZymes involved in fungal cell–wall biosynthesis, with many putative β-glucan enzymes and chitin synthases, and CAZymes for free carbohydrate metabolism were also present in the genome (Supplementary Table 6). These findings are consistent with the results of previous studies on CAZymes in the symbiotic fungal species *Penicillium*.

### Secondary metabolite biosynthetic gene clusters

**Fig. 5.**
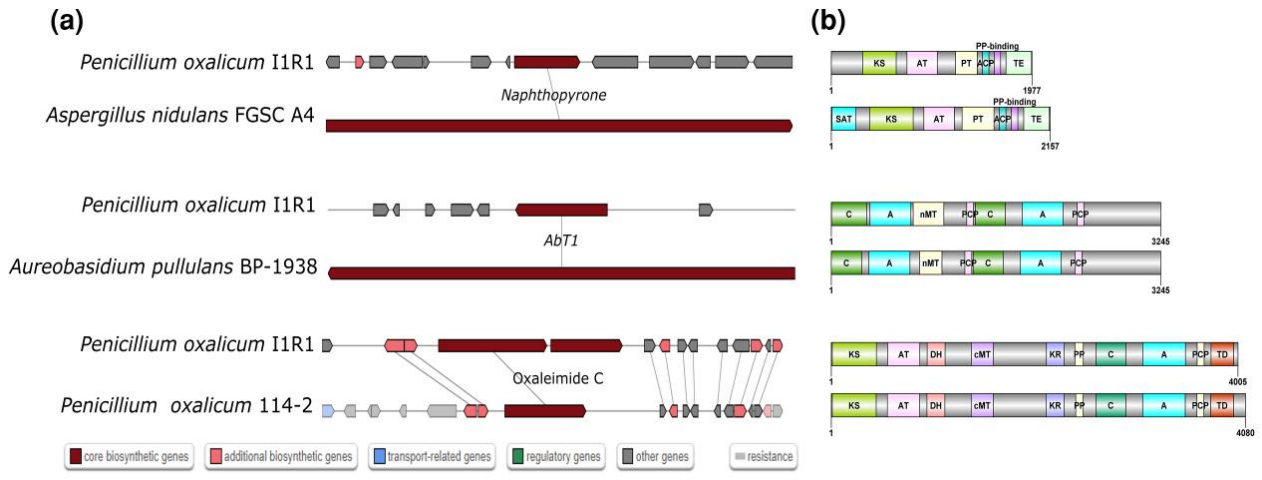
Typical smBGCs found in *P. oxalicum* I1R1 genomes compared with other reference smBGCs from MIBiG database. a) MIBiG comparison of protocluster to region. b) NRPS/PKS domain organization. A, adenylation domain; ACP, acyl-carrier protein domain; AT, acyltransferase domain; C, condensation domain linking an L-amino acid to a peptide ending with an L-amino acid; cMT, carbon methyltransferase; DH, dehydratase domain; KR, ketoreductase domain; KS, ketosynthase domain; nMT, nitrogen methyltransferase; PCP, peptidyl-carrier protein domain; PP, phosphopantetheine acyl-carrier protein group; PT, product template domain; SAT, Starter unit—ACP transacylase in aflatoxin biosynthesis; TD, terminal reductase domain; TE, thioesterase domain.

Genome mining of secondary metabolites was performed using antiSMASH. There were 36 found regions inside the *P. oxalicum* I1R1 genome that contained multiple types of smBGCs (see detail in Supplementary Table 7). Six different smBGCs types were detected, including betalactone, indole, NRPS, NRPS-like, T1PKS, and terpene. Almost all detected BGCs were unknown function clusters, showing the great potential of novel secondary metabolites discovery inside the *P. oxalicum* I1R1 genome. NRPS and NRPS-like were the most abundant types of smBGCs in *P. oxalicum* I1R1 genome with a total of 29 encoding clusters while there was only 1 smBGC encoding betalactone. Detected known clusters with similarity scores of at least 75% are an antitumor compound clavaric acid (100%), naphthopyrone (100%), an antifungal compound AbT1 (100%), and nidulanin A (75%). Yellow pigments (naphthopyrones) are commonly found in *Aspergillus* sp., *Fusarium* sp., and several plants (Song *et al*. 2004). These compounds were demonstrated to have anticancer and other specific bioactivities (Song *et al*. 2004). Genes encode for squalestatin (60%), a lead compound for the treatment of hypercholesterolemia by targeting squalene synthase (Sidebottom *et al*. 1992), and secalonic acids (50%) were also identified. The *P. oxalicum* species can be engineered to produce oxaleimide (Sato *et al*. 2017). In the I1R1 genome, we identified a BGC involving in oxaleimide producing with 60% genes showing similarity. According to the MIBiG comparison of the protocluster of I1R1 genome to the region of reference smBCGs, almost all the typical smBCGs adequately included the highly conserved core biosynthetic genes with the same organization of protein domain architecture (Fig. 5).

## Conclusion

By using the WGS approach and different bioinformatics tools and methods, this work provides a deeper insight of the novel *P. oxalicum* I1R1 genome. We present the whole-genome sequence and annotation of the I1R1 comprising eight chromosomes of 30.8 Mb size encoding 8,317 proteins, and an additional completed mitogenome of 26 kb. This fully completed *P. oxalicum* genome will provide a supplemental resource for further gene functional research as well as interspecies or intraspecies comparative analyses.

## Supplementary Material

jkac300_Supplementary_Data

## Data Availability

This Whole-Genome Shotgun project of *P. oxalicum* strain I1R1 has been deposited at DDBJ/ENA/GenBank under the accession CP093053-CP093060. The version described in this paper is version CP093053-CP093060. The reported assembly is associated with NCBI BioProject: PRJNA794314 and BioSample: SAMN24624473 within GenBank. Supplemental material available at G3 online.

## Notes

*Communicating editor: M. Sachs*
