# Supplementary material for: A highly quality genome sequence of Penicillium oxalicum species isolated from the root of Ixora chinensis in Vietnam
Source: G3 (Bethesda). 2022 Dec 1;13(2):jkac300. doi: 10.1093/g3journal/jkac300 (PMC9911084; doi:10.1093/g3journal/jkac300)

**Supplementary Table S1.** Summary of the *Penicillium oxalicum* I1R1 genome assembly

|  | **Telomeres** | | **Centromeres** |
| --- | --- | --- | --- |
|  | 5' | 3' |  |
| **Contigs** | | | |
| 1 | v | v | v |
| 2 | x | v | v |
| 3 | v | v | v |
| 4 | v | v | v |
| 5 | v | v | v |
| 6 | v | v | v |
| 7 | v | v | v |
| 8 | v | x | v |
| 9 | v | x | x |
| **Chromosome** | | | |
| 1 | v | v | v |
| 2 | v | v | v |
| 3 | v | v | v |
| 4 | v | v | v |
| 5 | v | v | v |
| 6 | v | v | v |
| 7 | v | v | v |
| 8 | v | x | v |
| **Note**: "v": detected; "x": non-detected | | | |

**Supplementary Table S2.** Global statistics of different assemblies from different *Penicillium oxalicum* strains available at DDBJ/ENA/GenBank

| **Parameters** | ***Penicillium oxalicum* strains** | | | | | | | | | |
| --- | --- | --- | --- | --- | --- | --- | --- | --- | --- | --- |
|  | **I1R1** | **CCTCC M 20211309** | **SGAir0226** | **HP7-1** | **SYJ-1** | **114-2** | **JU-A10-T** | **YT02** | **PM4501B** | **M7025A** |
| **Size (Mb)** | 30.8 | 30.54 | 30.68 | 30.66 | 29.84 | 30.18 | 30.69 | 31.25 | 29.8 | 30.2 |
| **Number of contigs/scafolds** | 8 | 9 | 20 | 263 | 313 | 345 | 482 | 674 | 215 | 176 |
| **Contig N50** | 3,834,910 | 4,444,577 | 2,487,861 | 526,863 | 168,146 | 157,054 | 154,88 | 92,919 | 541 | 612 |
| **Contig L50** | 4 | 3 | 5 | 21 | 53 | 63 | 66 | 101 | 19 | 17 |
| **BUSCO completeness** | 95 | 93.8 | 95 | 94.9 | 95 | 94.9 | 94.3 | 94.9 | 94.9 | 94.9 |
| **GC content (%)** | 50.6 | 50.69 | 50.6 | 50.6 | 50.8 | 50.6 | 50.6 | 49.8 | 50.5 | 50.5 |
| **Sequencing technology** | PacBio SEQUEL | Oxford Nanopore PromethION | PacBio RSII | Illumina HiSeq | Illumina HiSeq | 454 GS-FLX Titanium; ABI SOLiD | 454 GS-FLX Titanium; Illumina GAIIx | 454 GS-FLX Titanium; Illumina GAIIx; Illumina HiSeq | 454 GS-FLX Titanium; Illumina GAIIx; Illumina HiSeq | 454 GS-FLX Titanium; Illumina GAIIx; Illumina HiSeq |
| **Assembly method** | HGAP v. 4 | Unicycler software v. Version 0.4.8 | HGAP v. 3 | SOAPdenovo v. 2.04 | SOAPdenovo v. July-2013 | Newbler v. 2.3 | Newbler v. 2.3 | SOAPdenovo v. 2.0 | SPAdes v. 3.14.192 | SPAdes v. 3.14.192 |
| **Sequencing depth** | 400x | 100x | 40.7x | 108x | 40x | 28x | 14x | 440x | 292x | 291x |
| **GenBank assembly accession** | JAKCLI000000000 | GCA_021133555.1 | GCA_005546515.1 | GCA_001723175.2 | GCA_004153425.1 | GCA_000346795.1 | GCA_000383025.1 | GCA_002072455.1 | GCA_023624835.1 | GCA_023624855.1 |
| **Submission year** | 2022 | 2021 | 2019 | 2016 | 2019 | 2013 | 2013 | 2017 | 2022 | 2022 |

**Supplemental Table S3**. GO analysis of *Penicillium oxalicum* I1R1 genome

| **GO terms** | **Class** | **Number of genes** | **Percentage of genes (%)** | **Type** |
| --- | --- | --- | --- | --- |
| GO:0032991 | Cellular Component | 1678 | 43.7 | protein-containing complex |
| GO:0005623 |  | 3513 | 91.5 | cell |
| GO:0044464 |  | 3513 | 91.5 | cell part |
| GO:0044422 |  | 2121 | 55.2 | organelle part |
| GO:0043226 |  | 3038 | 79.1 | organelle |
| GO:0044425 |  | 872 | 22.7 | membrane part |
| GO:0016020 |  | 1192 | 31 | membrane |
| GO:0031974 |  | 959 | 25 | membrane-enclosed lumen |
| GO:0099080 |  | 44 | 1.1 | supramolecular complex |
| GO:0005576 |  | 114 | 3 | extracellular region |
| GO:0045202 |  | 1 | 0 | synapse |
| GO:0044456 |  | 1 | 0 | synapse part |
| GO:0055044 |  | 1 | 0 | symplast |
| GO:0030054 |  | 3 | 0.1 | cell junction |
| GO:0044421 |  | 19 | 0.5 | extracellular region part |
| GO:0009295 |  | 22 | 0.6 | nucleoid |
| GO:0003824 | Molecular Function | 1787 | 46.5 | catalytic activity |
| GO:0098772 |  | 215 | 5.6 | molecular function regulator |
| GO:0005215 |  | 276 | 7.2 | transporter activity |
| GO:0005488 |  | 1666 | 43.4 | binding |
| GO:0038024 |  | 2 | 0.1 | cargo receptor activity |
| GO:0140104 |  | 22 | 0.6 | molecular carrier activity |
| GO:0060089 |  | 22 | 0.6 | molecular transducer activity |
| GO:0045182 |  | 10 | 0.3 | translation regulator activity |
| GO:0005198 |  | 213 | 5.5 | structural molecule activity |
| GO:0016209 |  | 24 | 0.6 | antioxidant activity |
| GO:0140110 |  | 158 | 4.1 | transcription regulator activity |
| GO:0001076 |  | 26 | 0.7 | obsolete transcription factor activity, RNA polymerase II transcription factor binding |
| GO:0000989 |  | 30 | 0.8 | obsolete transcription factor activity, transcription factor binding |
| GO:0000988 |  | 58 | 1.5 | obsolete transcription factor activity, protein binding |
| GO:0000991 |  | 31 | 0.8 | obsolete transcription factor activity, core RNA polymerase II binding |
| GO:0000990 |  | 31 | 0.8 | obsolete transcription factor activity, core RNA polymerase binding |
| GO:0001167 |  | 2 | 0.1 | obsolete RNA polymerase I transcription factor activity, sequence-specific DNA binding |
| GO:0001129 |  | 5 | 0.1 | obsolete RNA polymerase II transcription factor activity, TBP-class protein binding, involved in preinitiation complex assembly |
| GO:0001132 |  | 5 | 0.1 | obsolete RNA polymerase II transcription factor activity, TBP-class protein binding |
| GO:0001191 |  | 7 | 0.2 | obsolete transcriptional repressor activity, RNA polymerase II transcription factor binding |
| GO:0001190 |  | 5 | 0.1 | obsolete transcriptional activator activity, RNA polymerase II transcription factor binding |
| GO:0001169 |  | 2 | 0.1 | obsolete transcription factor activity, RNA polymerase I CORE element sequence-specific binding |
| GO:0031386 |  | 6 | 0.2 | protein tag |
| GO:0009987 | Biological Process | 3362 | 87.6 | cellular process |
| GO:0032502 |  | 316 | 8.2 | developmental process |
| GO:0032501 |  | 43 | 1.1 | multicellular organismal process |
| GO:0071840 |  | 1695 | 44.1 | cellular component organization or biogenesis |
| GO:0065007 |  | 1539 | 40.1 | biological regulation |
| GO:0051179 |  | 1155 | 30.1 | localization |
| GO:0050789 |  | 1279 | 33.3 | regulation of biological process |
| GO:0048519 |  | 621 | 16.2 | negative regulation of biological process |
| GO:0050896 |  | 1058 | 27.6 | response to stimulus |
| GO:0023052 |  | 348 | 9.1 | signaling |
| GO:0008152 |  | 2887 | 75.2 | metabolic process |
| GO:0051704 |  | 324 | 8.4 | multi-organism process |
| GO:0022610 |  | 34 | 0.9 | biological adhesion |
| GO:0098743 |  | 6 | 0.2 | cell aggregation |
| GO:0048518 |  | 650 | 16.9 | positive regulation of biological process |
| GO:0040011 |  | 13 | 0.3 | locomotion |
| GO:0048511 |  | 2 | 0.1 | rhythmic process |
| GO:0022414 |  | 405 | 10.5 | reproductive process |
| GO:0000003 |  | 433 | 11.3 | reproduction |
| GO:0008283 |  | 6 | 0.2 | cell proliferation |
| GO:0002376 |  | 10 | 0.3 | immune system process |
| GO:0040007 |  | 213 | 5.5 | growth |
| GO:0015976 |  | 15 | 0.4 | carbon utilization |
| GO:0031657 |  | 1 | 0 | obsolete regulation of cyclin-dependent protein serine/threonine kinase activity involved in G1/S transition of mitotic cell cycle |
| GO:0031658 |  | 1 | 0 | obsolete negative regulation of cyclin-dependent protein serine/threonine kinase activity involved in G1/S transition of mitotic cell cycle |
| GO:0098754 |  | 53 | 1.4 | detoxification |
| GO:0001906 |  | 1 | 0 | cell killing |
| GO:0000456 |  | 1 | 0 | obsolete dimethylation involved in SSU-rRNA maturation |
| GO:0019740 |  | 25 | 0.7 | nitrogen utilization |
| GO:0043473 |  | 2 | 0.1 | pigmentation |
| GO:0044848 |  | 2 | 0.1 | biological phase |
| GO:0000903 |  | 2 | 0.1 | obsolete regulation of cell shape during vegetative growth phase |
| GO:0007610 |  | 1 | 0 | behavior |

**Supplemental Table S4.** Summary of major functional KEGG pathway annotation of predicted genes using GhostKOALA

| **Functional category** | ***Penicillium oxalicum I1R1*** |
| --- | --- |
| Annotated | 3805 entries (45.7%) annotated |
| **Metabolism** |  |
| (1) Carbohydrate metabolism | 347 |
| (2) Energy metabolism | 140 |
| (3) Lipid metabolism | 154 |
| (4) Nucleotide metabolism | 70 |
| (5) Amino acid metabolism | 270 |
| (6) Metabolism of other amino acids | 55 |
| (7) Glycan biosynthesis and metabolism | 93 |
| (8) Metabolism of cofactors and vitamins | 125 |
| (9) Metabolism of terpenoids and polyketides | 29 |
| (10) Biosynthesis of other secondary metabolites | 45 |
| (11) Xenobiotics biodegradation and metabolism | 68 |
| **Genetic Information Processing** |  |
| (12) Transcription | 141 |
| (13) Translation | 303 |
| (14) Folding, sorting and degradation | 248 |
| (15) Replication and repair | 144 |
| **Environmental Information Processing** |  |
| (16) Membrane transport |  |
| (a) ABC transporters | 8 |
| (b) Bacterial secretion system | 2 |
| (17) Signal transduction |  |
| (a) Two-component system | 19 |
| (b) Other | 407 |
| **Cellular Processes** |  |
| (18) Transport and catabolism | 331 |
| (19) Cell growth and death | 295 |
| (20) Cellular community |  |
| (a) Quorum sensing | 10 |
| (b) Biofilm formation | 3 |
| (21) Cell motility | 22 |

**Supplemental Table S5.** The Cytochromes P450 (CYPs) family in the *Penicillium oxalicum* I1R1 genome

| **Ko** | **Genes** |
| --- | --- |
| 00980 Metabolism of xenobiotics by cytochrome P450 (5) | |
| K00121 | g5598 |
| K00799 | g6813, g7404, g5043, g684, g851, g860, g2202, g3522, g3834, g4287, g4748 |
| K13299 | g5663 |
| K13953 | g6776, g5601, g1652, g5824, g3753, g4477 |
| K15303 | g3953 |
| 00982 Drug metabolism - cytochrome P450 (5) | |
| K00121 | g5598 |
| K00485 | g7697 |
| K00799 | g6813, g7404, g5043, g684, g851, g860, g2202, g3522, g3834, g4287, g4748 |
| K13299 | g5663 |
| K13953 | g6776, g5601, g1652, g5824, g3753, g4477 |

**Supplemental Table S6.** Putative carbohydrate-active enzymes (CAZymes) in *Penicillium oxalicu*m I1R1

|  | **Cazy family** | ***Penicillium oxalicum I1R1*** |
| --- | --- | --- |
| **Cellulose** | AA3 | 17 |
|  | AA8 | 2 |
|  | AA9 | 11 |
|  | GH1 | 8 |
|  | GH3 | 16 |
|  | GH5 | 19 |
|  | GH6 | 1 |
|  | GH7 | 3 |
|  | GH9 | 0 |
|  | GH12 | 4 |
|  | GH44 | 0 |
|  | GH45 | 0 |
|  | GT2 | 26 |
|  | Total | 107 |
| **Hemicellulase** | CBM13 | 3 |
|  | CE1 | 6 |
|  | CE4 | 8 |
|  | CE5 | 5 |
|  | CE7 | 0 |
|  | CE12 | 2 |
|  | GH2 | 8 |
|  | GH3 | 16 |
|  | GH5 | 19 |
|  | GH10 | 4 |
|  | GH11 | 5 |
|  | GH12 | 4 |
|  | GH16 | 14 |
|  | GH27 | 5 |
|  | GH29 | 0 |
|  | GH31 | 8 |
|  | GH35 | 4 |
|  | GH43 | 16 |
|  | GH51 | 4 |
|  | GH62 | 2 |
|  | GH74 | 0 |
|  | GH93 | 3 |
|  | GH95 | 1 |
|  | GH115 | 0 |
|  | GT8 | 8 |
|  | Total | 145 |
| **Pectin** | CE1 | 6 |
|  | CE8 | 11 |
|  | CE12 | 2 |
|  | GH2 | 8 |
|  | GH3 | 16 |
|  | GH28 | 13 |
|  | GH35 | 4 |
|  | GH43 | 16 |
|  | GH51 | 4 |
|  | GH78 | 6 |
|  | GH88 | 1 |
|  | GH93 | 3 |
|  | GH105 | 1 |
|  | PL1 | 4 |
|  | PL3 | 2 |
|  | PL4 | 3 |
|  | Total | 100 |
| **β-Glucans** | GH1 | 8 |
|  | GH3 | 16 |
|  | GH5 | 19 |
|  | GH7 | 3 |
|  | GH9 | 0 |
|  | GH12 | 4 |
|  | GH16 | 14 |
|  | GH17 | 5 |
|  | GH30 | 5 |
|  | GH72 | 7 |
|  | CBM18 | 5 |
|  | CBM43 | 1 |
|  | GT48 | 1 |
|  | Total | 88 |
| **Chitin** | GT2 | 26 |
|  | CE4 | 8 |
|  | GH18 | 23 |
|  | GH20 | 2 |
|  | Total | 59 |
| **α-Glucans + other FCWP** | GT5 | 0 |
|  | GH13 | 14 |
|  | GH71 | 9 |
|  | GH79 | 3 |
|  | Total | 26 |
| **Bacterial or animal polysaccharides** | PL8 | 0 |
|  | PL14 | 0 |
|  | GH79 | 3 |
|  | GH88 | 1 |
|  | Total | 4 |
| **Free Carbohydrates** | GH13 | 14 |
|  | GH15 | 3 |
|  | GH37 | 3 |
|  | GT3 | 3 |
|  | GT4 | 12 |
|  | GT20 | 9 |
|  | GT35 | 2 |
|  | Total | 46 |

**Supplemental Table S7**. Genome mining of secondary metabolite biosynthetic gene clusters of *Penicillium oxalicum* I1R1

| **Region** | **Type** | **From** | **To** | **Most similar known cluster** | **Type** | **Similarity** |
| --- | --- | --- | --- | --- | --- | --- |
| [Region 1.1](https://fungismash.secondarymetabolites.org/upload/fungi-aa92c48f-2dac-4d68-8dde-24d06092bbb2/index.html#r1c1) | [indole](https://docs.antismash.secondarymetabolites.org/glossary/#indole) | 739,373 | 760,639 | - | | |
| [Region 1.2](https://fungismash.secondarymetabolites.org/upload/fungi-aa92c48f-2dac-4d68-8dde-24d06092bbb2/index.html#r1c2) | [terpene](https://docs.antismash.secondarymetabolites.org/glossary/#terpene) | 1,263,318 | 1,282,227 | [clavaric acid](https://mibig.secondarymetabolites.org/go/BGC0001248/1) | Terpene | 100% |
| [Region 1.3](https://fungismash.secondarymetabolites.org/upload/fungi-aa92c48f-2dac-4d68-8dde-24d06092bbb2/index.html#r1c3) | [T1PKS](https://docs.antismash.secondarymetabolites.org/glossary/#t1pks) | 1,428,515 | 1,472,067 | [naphthopyrone](https://mibig.secondarymetabolites.org/go/BGC0000107/1) | Polyketide | 100% |
| [Region 1.4](https://fungismash.secondarymetabolites.org/upload/fungi-aa92c48f-2dac-4d68-8dde-24d06092bbb2/index.html#r1c4) | [T1PKS](https://docs.antismash.secondarymetabolites.org/glossary/#t1pks) | 2,169,670 | 2,211,298 | [secalonic acids](https://mibig.secondarymetabolites.org/go/BGC0001886/1) | Polyketide | 50% |
| [Region 1.5](https://fungismash.secondarymetabolites.org/upload/fungi-aa92c48f-2dac-4d68-8dde-24d06092bbb2/index.html#r1c5) | T1PKS,NRPS | 2,268,807 | 2,320,728 | [aspyridone A](https://mibig.secondarymetabolites.org/go/BGC0000959/1) | NRP + Polyketide:Iterative type I | 44% |
| [Region 1.6](https://fungismash.secondarymetabolites.org/upload/fungi-aa92c48f-2dac-4d68-8dde-24d06092bbb2/index.html#r1c6) | [NRPS-like](https://docs.antismash.secondarymetabolites.org/glossary/#nrps-like) | 4,640,046 | 4,683,066 | - | | |
| [Region 1.7](https://fungismash.secondarymetabolites.org/upload/fungi-aa92c48f-2dac-4d68-8dde-24d06092bbb2/index.html#r1c7) | [NRPS-like](https://docs.antismash.secondarymetabolites.org/glossary/#nrps-like) | 4,993,426 | 5,023,261 | - | | |
| [Region 1.8](https://fungismash.secondarymetabolites.org/upload/fungi-aa92c48f-2dac-4d68-8dde-24d06092bbb2/index.html#r1c8) | [NRPS-like](https://docs.antismash.secondarymetabolites.org/glossary/#nrps-like) | 5,471,701 | 5,514,708 | - | | |
| [Region 1.9](https://fungismash.secondarymetabolites.org/upload/fungi-aa92c48f-2dac-4d68-8dde-24d06092bbb2/index.html#r1c9) | [terpene](https://docs.antismash.secondarymetabolites.org/glossary/#terpene) | 5,744,528 | 5,765,855 | - | | |
| [Region 2.1](https://fungismash.secondarymetabolites.org/upload/fungi-aa92c48f-2dac-4d68-8dde-24d06092bbb2/index.html#r2c1) | [NRPS](https://docs.antismash.secondarymetabolites.org/glossary/#nrps) | 457,45 | 509,726 | - | | |
| [Region 2.2](https://fungismash.secondarymetabolites.org/upload/fungi-aa92c48f-2dac-4d68-8dde-24d06092bbb2/index.html#r2c2) | [NRPS-like](https://docs.antismash.secondarymetabolites.org/glossary/#nrps-like) | 3,462,477 | 3,507,039 | - | | |
| [Region 2.3](https://fungismash.secondarymetabolites.org/upload/fungi-aa92c48f-2dac-4d68-8dde-24d06092bbb2/index.html#r2c3) | [indole](https://docs.antismash.secondarymetabolites.org/glossary/#indole) | 4,494,425 | 4,515,711 | - | | |
| [Region 2.4](https://fungismash.secondarymetabolites.org/upload/fungi-aa92c48f-2dac-4d68-8dde-24d06092bbb2/index.html#r2c4) | [terpene](https://docs.antismash.secondarymetabolites.org/glossary/#terpene) | 4,838,120 | 4,859,734 | [squalestatin S1](https://mibig.secondarymetabolites.org/go/BGC0001839/1) | Terpene | 60% |
| [Region 3.1](https://fungismash.secondarymetabolites.org/upload/fungi-aa92c48f-2dac-4d68-8dde-24d06092bbb2/index.html#r3c1) | [NRPS](https://docs.antismash.secondarymetabolites.org/glossary/#nrps) | 1,186,157 | 1,229,857 | - | | |
| [Region 3.2](https://fungismash.secondarymetabolites.org/upload/fungi-aa92c48f-2dac-4d68-8dde-24d06092bbb2/index.html#r3c2) | [NRPS](https://docs.antismash.secondarymetabolites.org/glossary/#nrps) | 1,590,527 | 1,649,070 | [nidulanin A](https://mibig.secondarymetabolites.org/go/BGC0001699/1) | Polyketide | 75% |
| [Region 3.3](https://fungismash.secondarymetabolites.org/upload/fungi-aa92c48f-2dac-4d68-8dde-24d06092bbb2/index.html#r3c3) | [terpene](https://docs.antismash.secondarymetabolites.org/glossary/#terpene) | 1,728,636 | 1,751,698 | - | | |
| [Region 3.4](https://fungismash.secondarymetabolites.org/upload/fungi-aa92c48f-2dac-4d68-8dde-24d06092bbb2/index.html#r3c4) | [NRPS-like](https://docs.antismash.secondarymetabolites.org/glossary/#nrps-like) | 2,181,327 | 2,225,160 | - | | |
| [Region 4.1](https://fungismash.secondarymetabolites.org/upload/fungi-aa92c48f-2dac-4d68-8dde-24d06092bbb2/index.html#r4c1) | [NRPS](https://docs.antismash.secondarymetabolites.org/glossary/#nrps) | 349,401 | 389,814 | - | | |
| [Region 4.2](https://fungismash.secondarymetabolites.org/upload/fungi-aa92c48f-2dac-4d68-8dde-24d06092bbb2/index.html#r4c2) | [T1PKS](https://docs.antismash.secondarymetabolites.org/glossary/#t1pks) | 1,830,784 | 1,871,423 | - | | |
| [Region 4.3](https://fungismash.secondarymetabolites.org/upload/fungi-aa92c48f-2dac-4d68-8dde-24d06092bbb2/index.html#r4c3) | T1PKS,NRPS | 2,176,640 | 2,230,063 | [oxaleimide C](https://mibig.secondarymetabolites.org/go/BGC0001724/1) | NRP + Polyketide | 60% |
| [Region 4.4](https://fungismash.secondarymetabolites.org/upload/fungi-aa92c48f-2dac-4d68-8dde-24d06092bbb2/index.html#r4c4) | [NRPS](https://docs.antismash.secondarymetabolites.org/glossary/#nrps) | 2,343,709 | 2,392,550 | - | | |
| [Region 4.5](https://fungismash.secondarymetabolites.org/upload/fungi-aa92c48f-2dac-4d68-8dde-24d06092bbb2/index.html#r4c5) | T1PKS,NRPS | 2,886,489 | 2,938,789 | - | | |
| [Region 4.6](https://fungismash.secondarymetabolites.org/upload/fungi-aa92c48f-2dac-4d68-8dde-24d06092bbb2/index.html#r4c6) | [NRPS-like](https://docs.antismash.secondarymetabolites.org/glossary/#nrps-like) | 3,417,762 | 3,451,745 | - | | |
| [Region 4.7](https://fungismash.secondarymetabolites.org/upload/fungi-aa92c48f-2dac-4d68-8dde-24d06092bbb2/index.html#r4c7) | [NRPS](https://docs.antismash.secondarymetabolites.org/glossary/#nrps) | 3,716,278 | 3,766,078 | [AbT1](https://mibig.secondarymetabolites.org/go/BGC0000307/1) | NRP | 100% |
| [Region 5.1](https://fungismash.secondarymetabolites.org/upload/fungi-aa92c48f-2dac-4d68-8dde-24d06092bbb2/index.html#r5c1) | [terpene](https://docs.antismash.secondarymetabolites.org/glossary/#terpene) | 412,082 | 433,303 | - | | |
| [Region 5.2](https://fungismash.secondarymetabolites.org/upload/fungi-aa92c48f-2dac-4d68-8dde-24d06092bbb2/index.html#r5c2) | [betalactone](https://docs.antismash.secondarymetabolites.org/glossary/#betalactone) | 1,075,645 | 1,107,081 | - | | |
| [Region 5.3](https://fungismash.secondarymetabolites.org/upload/fungi-aa92c48f-2dac-4d68-8dde-24d06092bbb2/index.html#r5c3) | NRPS,indole | 2,233,992 | 2,323,314 | [paraherquamide](https://mibig.secondarymetabolites.org/go/BGC0000819/1) | NRP + Alkaloid | 20% |
| [Region 5.4](https://fungismash.secondarymetabolites.org/upload/fungi-aa92c48f-2dac-4d68-8dde-24d06092bbb2/index.html#r5c4) | indole,NRPS | 3,181,661 | 3,254,239 | [histidyltryptophanyldiketopiperazine / dehydrohistidyltryptophanyldiketopiperazine / roquefortine D / roquefortine C / glandicoline A / glandicoline B / meleagrine](https://mibig.secondarymetabolites.org/go/BGC0000420/1) | NRP | 28% |
| [Region 5.5](https://fungismash.secondarymetabolites.org/upload/fungi-aa92c48f-2dac-4d68-8dde-24d06092bbb2/index.html#r5c5) | [NRPS-like](https://docs.antismash.secondarymetabolites.org/glossary/#nrps-like) | 3,446,260 | 3,470,389 | - | | |
| [Region 5.6](https://fungismash.secondarymetabolites.org/upload/fungi-aa92c48f-2dac-4d68-8dde-24d06092bbb2/index.html#r5c6) | [NRPS](https://docs.antismash.secondarymetabolites.org/glossary/#nrps) | 3,522,731 | 3,583,112 | - | | |
| [Region 5.7](https://fungismash.secondarymetabolites.org/upload/fungi-aa92c48f-2dac-4d68-8dde-24d06092bbb2/index.html#r5c7) | NRPS,T1PKS | 3,626,881 | 3,678,928 | [ilicicolin H](https://mibig.secondarymetabolites.org/go/BGC0002035/1) | NRP + Polyketide | 33% |
| [Region 6.1](https://fungismash.secondarymetabolites.org/upload/fungi-aa92c48f-2dac-4d68-8dde-24d06092bbb2/index.html#r6c1) | NRPS,T1PKS | 58,368 | 139,939 | - | | |
| [Region 6.2](https://fungismash.secondarymetabolites.org/upload/fungi-aa92c48f-2dac-4d68-8dde-24d06092bbb2/index.html#r6c2) | [NRPS-like](https://docs.antismash.secondarymetabolites.org/glossary/#nrps-like) | 283,417 | 323,363 | - | | |
| [Region 6.3](https://fungismash.secondarymetabolites.org/upload/fungi-aa92c48f-2dac-4d68-8dde-24d06092bbb2/index.html#r6c3) | [NRPS](https://docs.antismash.secondarymetabolites.org/glossary/#nrps) | 1,613,915 | 1,665,405 | - | | |
| [Region 8.1](https://fungismash.secondarymetabolites.org/upload/fungi-aa92c48f-2dac-4d68-8dde-24d06092bbb2/index.html#r8c1) | [NRPS-like](https://docs.antismash.secondarymetabolites.org/glossary/#nrps-like) | 333,164 | 370,224 | - | | |
| [Region 8.2](https://fungismash.secondarymetabolites.org/upload/fungi-aa92c48f-2dac-4d68-8dde-24d06092bbb2/index.html#r8c2) | [NRPS](https://docs.antismash.secondarymetabolites.org/glossary/#nrps) | 718,05 | 766,189 | - | | |

Note: "-" unknown

**Supplemental Figure S1.** Morphological characterization of *Penicillium oxalicum* I1R1 from Ixora chinensis. (A) White colony type. (B) Conidiophore branching patterns structures.


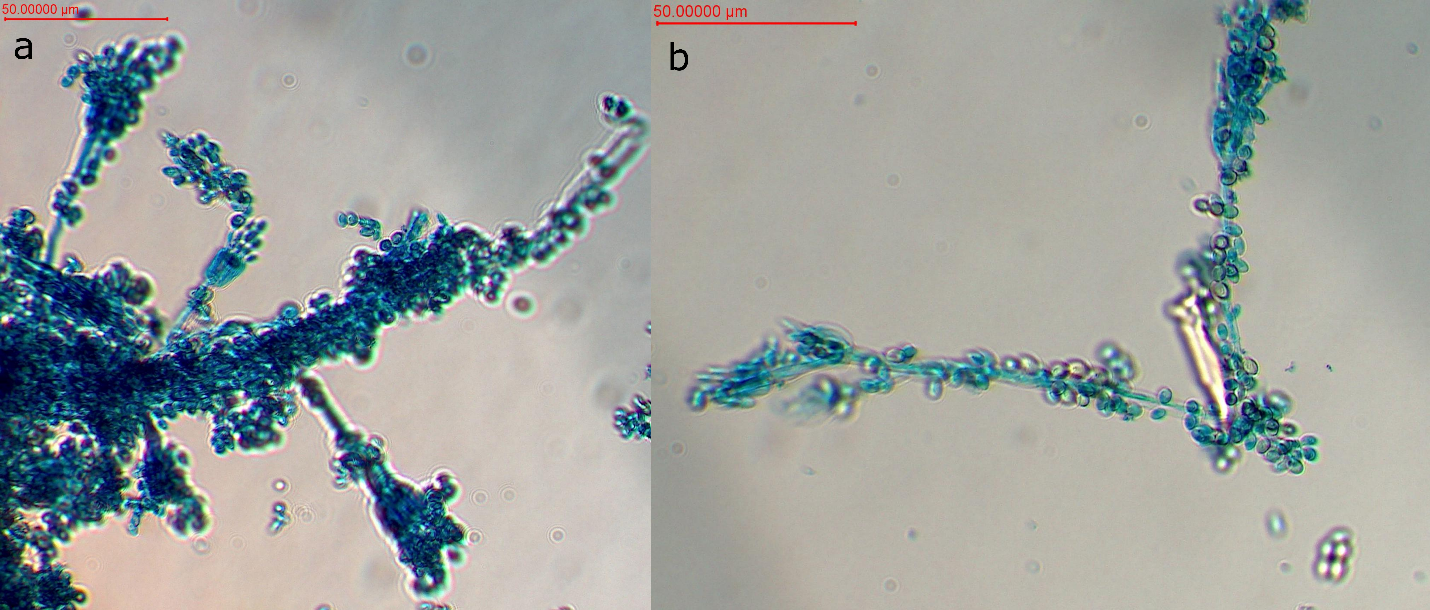


**Supplemental Figure S2: Clusters of orthologous groups (COGs, A) and Gene Ontology (GO, B) classification of *Penicillium oxalicum* I1R1 protein genes.** (A) A total of 7,226 produced functional annotations were among the 25 categories. The Y-axis shows the number of protein-coding genes in each COG term. (B) The GO classification map includes the numbers of genes and their involvement in cellular components, molecular functions, and biological processes. The numbers on top of the bars show the number of genes assigned to each GO term.


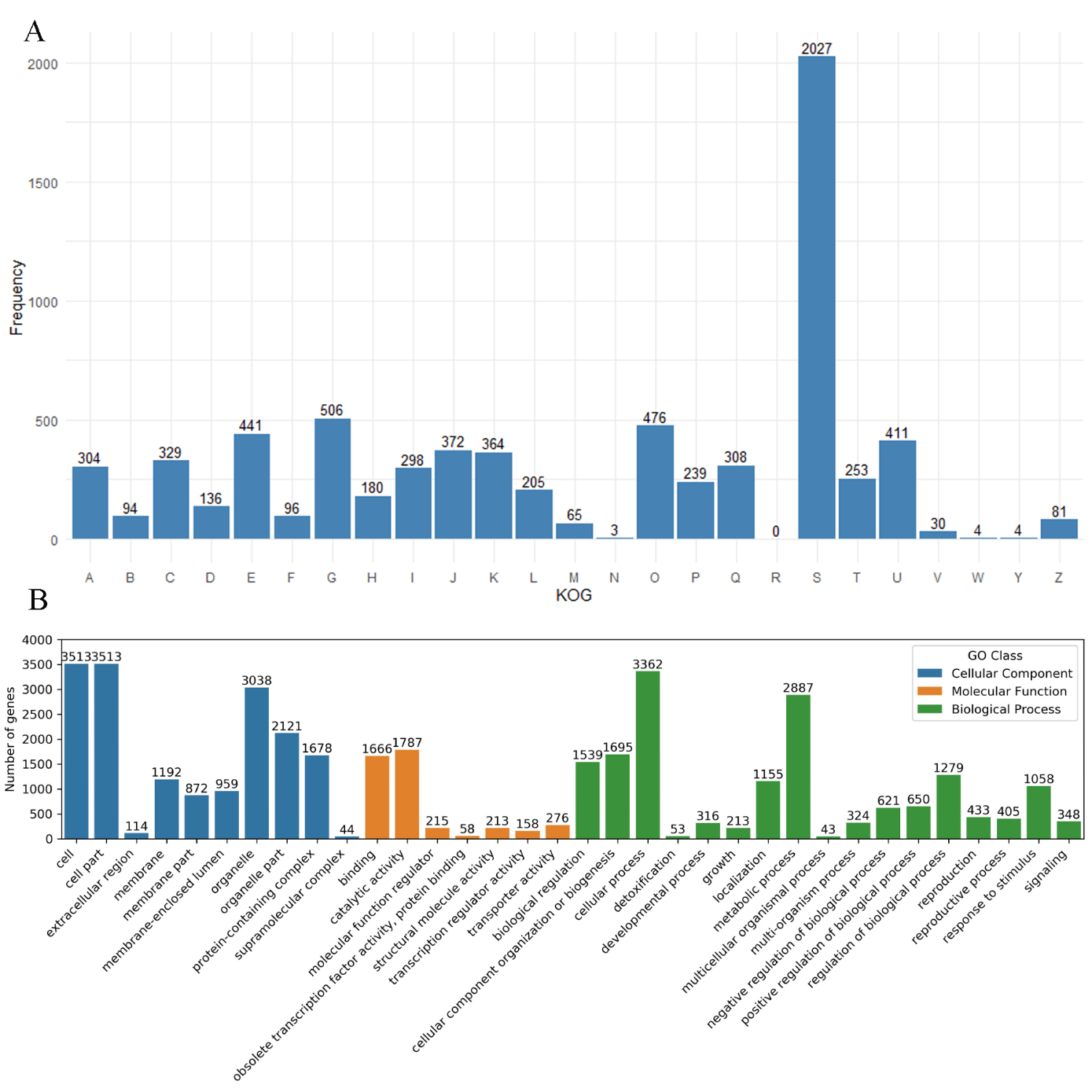


**Supplemental Figure S3: Functional KEGG pathway annotation of predicted genes**. The KEGG pathway map includes the numbers of genes and their involvement in metabolism, genetic information processing, environmental information processing, and cellular processes. The numbers on top of the bars show the number of genes assigned to each KEGG pathway term.


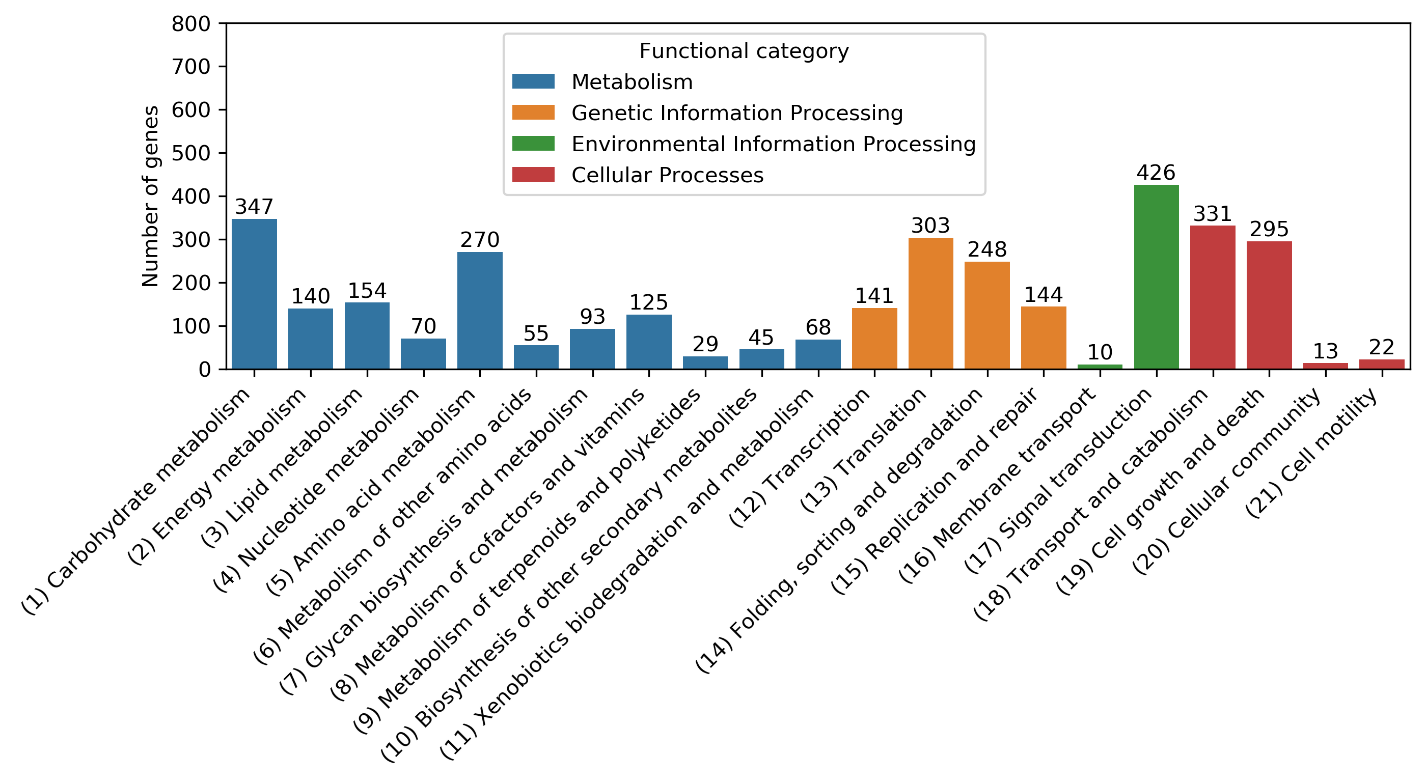

Supplement: jkac300_Supplementary_Data [file jkac300_supplementary_data.docx]
